# Comparison of neoadjuvant immunotherapy versus routine neoadjuvant therapy for patients with locally advanced esophageal cancer: A systematic review and meta-analysis

**DOI:** 10.3389/fimmu.2023.1108213

**Published:** 2023-03-23

**Authors:** Hao Qin, Futao Liu, Yaozhong Zhang, Yuxiang Liang, Yuan Mi, Fan Yu, Haidi Xu, Kuankuan Li, Chenxi Lin, Lei Li, Ziqiang Tian, Lei Wang

**Affiliations:** ^1^ Emergency Department, The Fourth Hospital of Hebei Medical University, Shijiazhuang, China; ^2^ Department of Thoracic Surgery, The Fourth Hospital of Hebei Medical University, Shijiazhuang, China

**Keywords:** esophageal cancer, neoadjuvant, immune checkpoint inhibitor, chemotherapy, chemoradiotherapy, pathological complete response, meta-analysis

## Abstract

**Background:**

The neoadjuvant use of immune checkpoint inhibitor combined with chemotherapy (nICT) or chemoradiotherapy (nICRT) in locally advanced esophageal cancer (EC) is currently an area of active ongoing research. Therefore, we carried out a comprehensive meta-analysis to compare the efficacy and safety of the new strategy with routine neoadjuvant strategy, which included neoadjuvant chemotherapy (nCT) and neoadjuvant chemoradiotherapy (nCRT).

**Patients and methods:**

MEDLINE (via PubMed), Embase (via OVID), ISI Web of Science database and Cochrane Library were included. And, all of them were searched for eligible studies between January, 2000 and February, 2023. The pathological complete response (pCR) and major pathological response (MPR) were primary outcome of our study. The second outcome of interest was R0 resection rate. Odds ratio (OR) and associated 95% CI were used as the effect indicators comparing the safety and efficiency of the neoadjuvant immunotherapy with the routine neoadjuvant therapy. Fixed-effect model (Inverse Variance) or random-effect model (Mantel-Haenszel method) was performed depending on the statistically heterogeneity.

**Results:**

There were eight trials with 652 patients were included in our meta-analysis. The estimated pCR rate was higher in the neoadjuvant immunotherapy group (OR =1.86; 95% CI, 1.25–2.75; I^2^ = 32.8%, *P*=0.166). The different results were found in the esophageal squamous cell carcinoma (ESCC) and esophageal adenocarcinoma (EAC) subgroups, the estimated OR was 2.35 (95%CI, 1.00–2.72; I^2^ = 30.9%, *P*=0.215) in the EAC subgroup, and 2.35 (95% CI, 1.20–4.54; I^2^ = 45.3%, *P*=0.161) in the ESCC subgroup, respectively. The neoadjuvant immunotherapy also showed the advantage in the MPR rates (OR =2.66; 95% CI, 1.69–4.19; I^2^ = 24.3%, *P*=0.252). There was no obvious difference between the neoadjuvant immunotherapy and routine neoadjuvant therapy with respect to surgical resection rate, R0 resection rate, surgical delay rate; while more treatment-related adverse events were observed for the neoadjuvant immunotherapy for pneumonitis/pneumonia (OR=3.46, 95% CI, 1.31–9.16; I^2^ = 67.3%, *P*=0.005) and thyroid dysfunction (OR=4.69, 95% CI, 1.53–14.36; I^2^ = 56.5%, *P*=0.032).

**Conclusion:**

The pooled correlations indicated that the neoadjuvant immunotherapy (both nICT and nICRT) could significantly increase the rates of pCR and MPR, compared with routine neoadjuvant therapy (both nCT and nCRT) in the treatment of locally advanced EC. The neoadjuvant immunotherapy and routine neoadjuvant therapy were with acceptable toxicity. However, randomized studies with larger groups of patients need to performed to confirm these results.

**Systematic review registration:**

https://www.crd.york.ac.uk/prospero/, identifier CRD42020155802.

## Introduction

Esophageal cancer is one of the deadliest cancers. As the eighth most commonly diagnosed cancer worldwide, there were 544,000 cancer-related deaths of EC in 2020, ranked sixth of cancer-related mortality (1). According to the latest data of China National Cancer Center, esophageal cancer ranked the sixth and the mortality ranked the fourth. EC includes two main histological subtypes, EAC and ESCC. The ESCC accounts for about 90% of esophageal cancer patients. As an aggressive cancer, the five-year survival rate of ESCC was just 35–45%, and the EAC was even lower.

Surgery remains the mainstay for ESCC or EAC, but surgery alone did not show satisfactory clinical data. Some studies showed that neoadjuvant therapy was the most effective strategy in improving survival of resectable esophageal cancer (2, 3). At present, the neoadjuvant therapy is widely applied to improve long-term survival rate in clinical trials. There were two randomized controlled trials (RCTs) demonstrated the neoadjuvant CRT (nCRT) was an effective and safe therapy strategy for locally advanced EC, NEOCRTEC5010 (nCRT for ESCC) and CROSS (nCRT for EC) (4, 5). In addition, the neoadjuvant chemotherapy (nCT) was another standard treatment for locally advanced ESCC patients, especially in Japan (6). However, the 5-year overall survival rate of nCRT or nCT was only 47%, and 3-year disease free survival was about 49%.

Immune checkpoint inhibitors (ICIs) combined with chemotherapy, as first line, obviously improved survival data of patients with advanced/metastatic esophageal cancer (7–11). The efficacy of neoadjuvant ICIs combined with nCT has been previously reported in esophageal cancer (12, 13). Recent meta-analyses have demonstrated the neoadjuvant ICIs combined with nCT or nCRT had promising clinical result and acceptable safety outcomes for patients with locally advanced EC (14–17). Nevertheless, there was no any meta-analyses comparing neoadjuvant ICIs combined with nCT or nCRT with routine neoadjuvant therapy, which included nCRT and nCT.

We summarized the recent studies and carried out this systematic review and meta-analysis to compare the efficacy and safety of the neoadjuvant immunotherapy with the routine neoadjuvant therapy followed by esophagectomy for patients with locally advanced EC.

## Methods

This study was performed according to the Preferred Reporting Items for Systematic Reviews and Meta-analyses (PRISMA) and Meta-analysis of Observational Studies in Epidemiology (MOOSE) reporting guidelines (18, 19) (checklists presented in the **Supplement**). This systematic review and meta-analysis were registered at International Prospective Register of Systematic Reviews (CRD42020155802).

### Search strategy and study selection

We identified eligible studies comparing the neoadjuvant immunotherapy with routine neoadjuvant therapy in the treatment of locally advanced EC in the MEDLINE (via PubMed), Embase (via OVID), ISI Web of Science database and Cochrane Library, between January, 2000 and February, 2023. The language was limited to English. The following search terms or keywords were used: esophageal cancer (MeSH) OR esophageal squamous cell carcinoma OR esophageal adenocarcinoma AND neoadjuvant OR preoperative AND programmed cell death 1 (PD-1) OR programmed cell death ligand 1 (PD-L1) OR immunotherapy (**Supplement Table S2**). The last search was conducted on February 6, 2023. All titles and abstracts were screened and reviewed carefully.

Two authors (H.D.X. and K.L.) independently retrieved the available literature to identify the eligible studies. The studies were chosen on the basis of the following criteria: (a) studies only including patients with esophagus cancer or esophagogastric cancer; (b) the primary efficacy outcomes were pathological complete response rate; complete (R0) tumor resection rate; adverse events of neoadjuvant treat; (c) Randomized Controlled Trials (RCTs) or Retrospective experiments comparing neoadjuvant ICIs combined with nCT or nCRT for treating EC and (d) The experimental design met the requirements and included patients with ESCC and EAC. Exclusion criteria were as the following criteria: (a) studies reporting incomplete or inconsistent outcomes; and (b) duplicate studies, studies reporting animal experiments, case reports, cohort studies, and review articles.

### Data collection and quality assessment

Data extraction was respectively and carefully performed by two reviewers (H.D.X. and K.L.). The following information was collected: first author, year of publication, region, characteristics of the study population (number, sex and age), TNM stage, treatment therapy, adverse events of neoadjuvant therapies, postoperative complications, and pathological response. If the HR and its 95% CI were not directly provided in the original articles, the extracted survival information and the published risk table were used to reconstruct the survival curve for each included study using the method of David (20). The extraction of information was repeated if there were apparent discrepancies. Reviewers would contact the corresponding authors of the studies to access relevant data to analysis, when no sufficient data in publications were extracted. The methodological quality was assessed by reviewers (H.D.X. and K.L.) using the Newcastle-Ottawa Scale (NOS). Moderate quality was defined as 4-6 scores, and 7-9 scores was high quality. An additional adjudicator (L.W.) would be invited into the discussion to resolve the discrepancies between the reviewers. To ensure that patients were not counted several times, we selected data with the largest number of participants if a medical database was used by multiple studies in adjacent time periods and the number of patients were similar.

### Outcome measures

The neoadjuvant immunotherapy comprised neoadjuvant immune checkpoint inhibitor in combination with chemotherapy (nICT) and neoadjuvant immune checkpoint inhibitor in combination with chemoradiotherapy (nICRT). The routine neoadjuvant therapy included neoadjuvant chemotherapy (nCT) and neoadjuvant chemoradiotherapy (nCRT).

The pathological TNM stage was staged according to the 8th edition American Joint Committee on Cancer/Union for International Cancer Control staging system (21). We used Response Evaluation Criteria In Solid Tumours guideline version 1.13 system to classify regressive changes after neoadjuvant treatment based on histopathological results to reveal prognostic information (22). The treatment related adverse events (TRAEs) were assessed by Common Terminology Criteria for Adverse Events, version 4.0 (23).

Pathologic complete response (pCR) was defined as no evidence of residual tumor cells of the complete resected tumor specimen of neoadjuvant therapy and resection. The major pathological response (MPR) was defined as less than 10% of residual tumor cells. In the present study, the pCR and MPR rates were considered to be the primary outcomes. R0 resection was defined as a microscopically margin-negative resection without microscopic tumor on the primary tumor bed. The R0 surgical resection rate was set as the secondary outcome for comparing neoadjuvant immunotherapy plus chemotherapy with chemotherapy alone for patients.

### Statistical analysis

The primary outcome of interest was pathologic response (pCR and MPR). The second outcome of interest was R0 resection rate. Odds ratio (OR) and associated 95% CI were used as the effect indicators comparing the safety and efficiency of the neoadjuvant immunotherapy with the routine neoadjuvant therapy. To minimize the influence of recall and selection bias that occur in retrospective studies, we performed stratified analyses to assess the association in all cohort studies. The heterogeneity between studies was evaluated with Q and I^2^ statistics (24). The results were calculated using a random-effect model (Mantel-Haenszel method) when statistically heterogeneity (I^2^ >50%) between studies were found. If low heterogeneity (I^2^ ≤50%) was between studies fixed-effect model (Inverse Variance) was performed.

Sensitivity analysis, subgroup analysis and meta-regression were all performed to explore the sources of heterogeneity. The potential publication bias was further validated by the Egger’s and Begg’s test (25). All statistical analyses were two sides; and P value less than 0.05 was considered statistically significant. Statistical analysis was performed using the STATA version 15.0 (Stata Corp LP, College Station, Texas, USA).

## Results

### Characteristics of included studies

**Figure 1:**
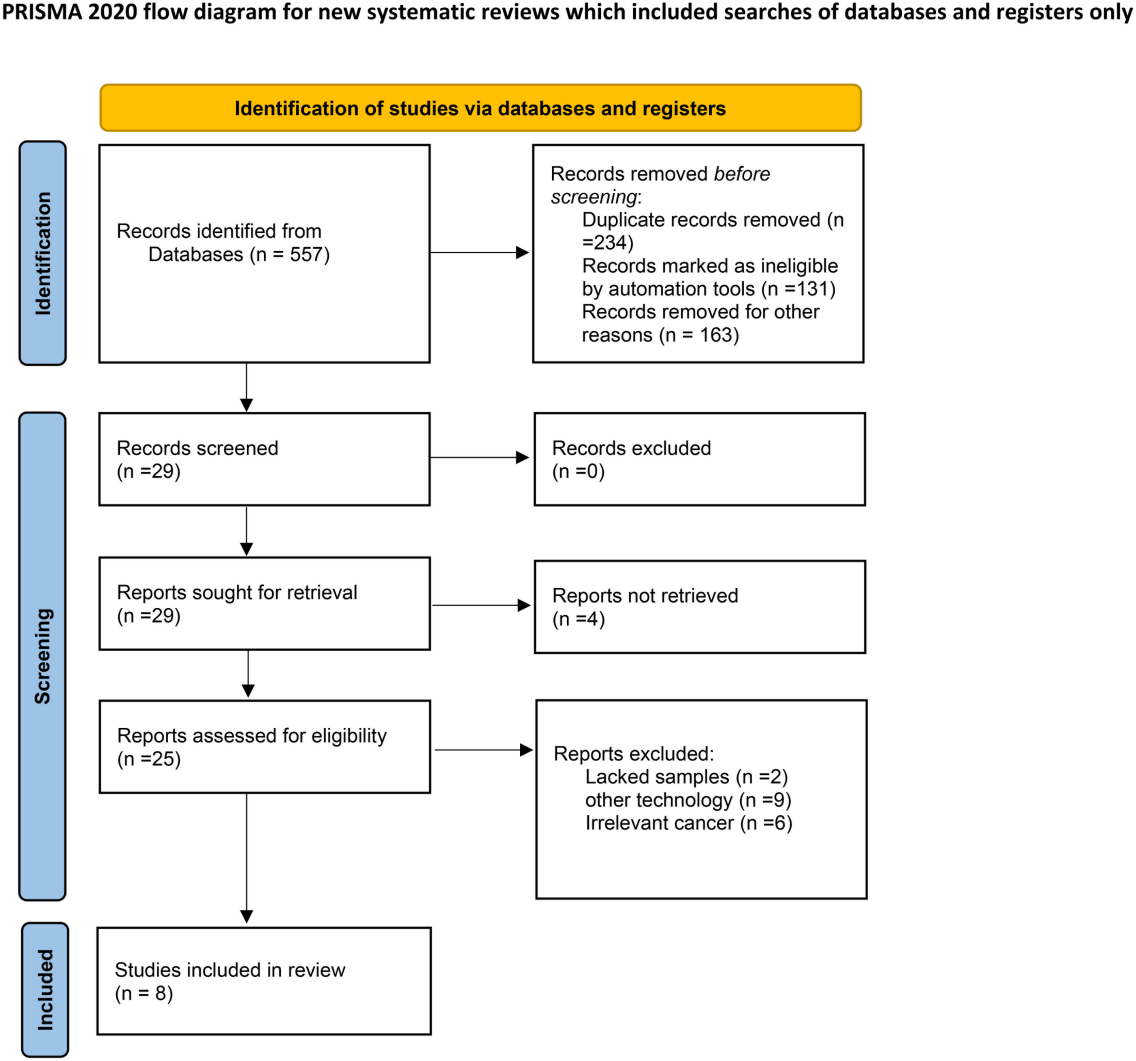
Flow diagram of included studies for this meta-analysis.

After reviewing 557 publications found using the predefined search terms. All investigators finally agreed to include eight eligible studies (26–33) with 652 patients in our meta-analysis (**Table 1**). The PRISMA flow chart of this meta-analysis was shown in **Figure 1**. Among them, five studies were conducted on esophageal squamous cell carcinoma (ESCC) (26–28, 31, 32), and the other three addressed esophageal adenocarcinoma cancer (EAC) (29, 30, 33). About the neoadjuvant strategies, there were four studies that studied nICT vs nCT (26, 28, 31, 33), two studies that studied nICT vs nCRT (27, 32), two studies that studied nICRT vs nCRT (29, 30). The sample size was ranged from 47 to 168. The Newcastle-Ottawa scores are presented in the **Supplement Table S2**.

**Table 1:** Characteristics of included studies for the meta-analyses.

Study	Country	Enrolled patients				Intervention	ICI	Neoadjuvant cycle	NCT or ChiCTR identifier
		Sample size, No.	Male, No. (%)	Clinical stage	Histological type				
Bingjiang Huang et al, 2021 (26)	China	54	51 (94.4%)	cT2-4N1-3M0	ESCC	nICT vs nCT	pembrolizumab	2	ChiCTR2000035079
Zhinuan Hong et al, 2022 (27)	China	87	68 (78.2%)	cT1N1-3M0 or cT2-4aN 0-3M0	ESCC	nICT vs nCRT	sintilimab pembrolizumab toripalimab camrelizumab	2-4	NR
Shaowu Jing et al, 2022 (28)	China	94	63 (67.0%)	cT3-4aN0-2M0	ESCC	nICT vs nCT	sintilimab pembrolizumab toripalimab camrelizumab	1-3	NR
Smita Sihag et al, 2021 (29)	USA	168	146 (86.9%)	NR	EAC	nICRT vs nCRT	durvalumab	2	NCT02962063
Tom van don Ende et al, 2021 (30)	Netherlands	80	71 (88.7%)	NR	EAC	nICRT vs nCRT	atezolizumab	5	NCT03087864
Zhinuan Hong et al, 2021 (31)	China	122	101 (82.8%)	cT1N1-3 M0 or cT2-4aN 0-3M0	ESCC	nICT vs nCT	sintilimab pembrolizumab camrelizumab	2-4	ChiCTR2100045659
Jiahan Cheng et al, 2022 (32)	China	149	123 (82.6%)	cT2-4N1-3M0	ESCC	nICT vs nCRT	sintilimab pembrolizumab camrelizumab toripalimab tislelizumab	2-4	NR
Xuewei Ding et al, 2023 (33)	China	47	NR	NR	EAC	nICT vs nCT	sintilimab	3	NCT04982939

ESCC, esophageal squamous cell carcinoma; EAC, esophageal adenocarcinoma; nICT, neoadjuvant immune checkpoint inhibitor in combination with chemotherapy; nICRT, neoadjuvant immune checkpoint inhibitor in combination with chemoradiotherapy; nCT, neoadjuvant chemotherapy; nCRT, neoadjuvant chemoradiotherapy; NR, not reported.

### pCR and MPR

**Figure 2:**
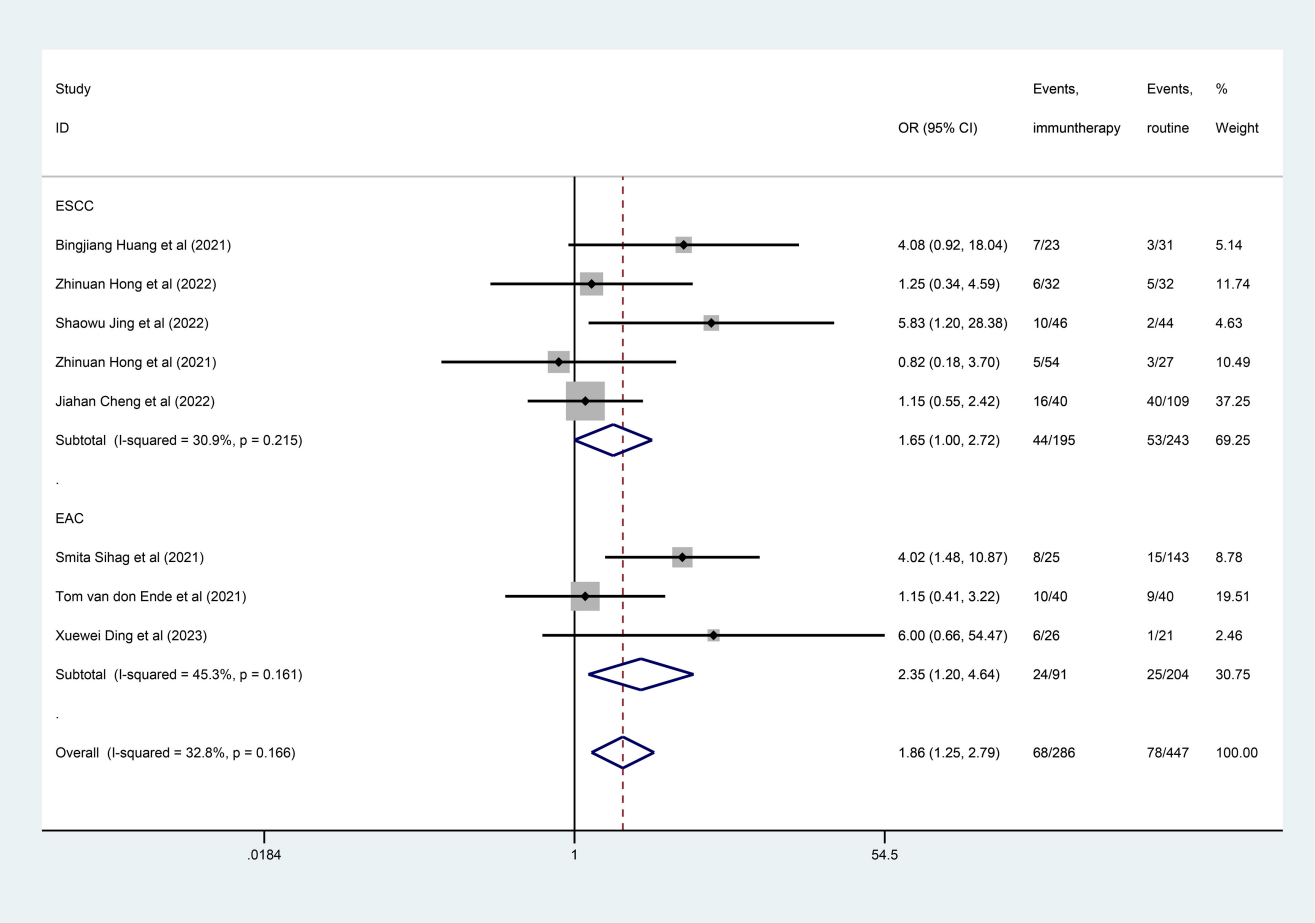
Forest plot of pathological complete response (pCR).

Eight studies (26–33) were included in the pCR meta-analysis. Due to the heterogeneity between studies (I^2^ = 32.8%, *P*=0.166), the data from the subgroups within a single study was pooled using a fixed-effect model. The estimated pCR rate was higher in the neoadjuvant immunotherapy group, including nICT and nICRT (OR =1.86; 95% CI, 1.25–2.79; **Figure 2**). As to the difference of the histologic subtypes, the studies were divided into two subgroups (the EAC group and the ESCC group). However, the different results were found in the ESCC and EAC subgroups, the estimated OR was 2.35 (95%CI, 1.20–4.64) in the EAC subgroup, and 1.65 (95% CI, 1.00–2.72) in the ESCC subgroup. The heterogeneity of two subgroups were (I^2^ = 45.3%, *P*=0.161) and (I^2^ = 30.9%, *P*=0.215), respectively. Interestingly, we found the common result (OR=1.93, 95% CI, 1.08–3.46; I^2^ = 57.5%, *P*=0.094) (see **Supplementary Material 3: Figure S1**), when we deleted all studies included nCRT.

**Figure 3:**
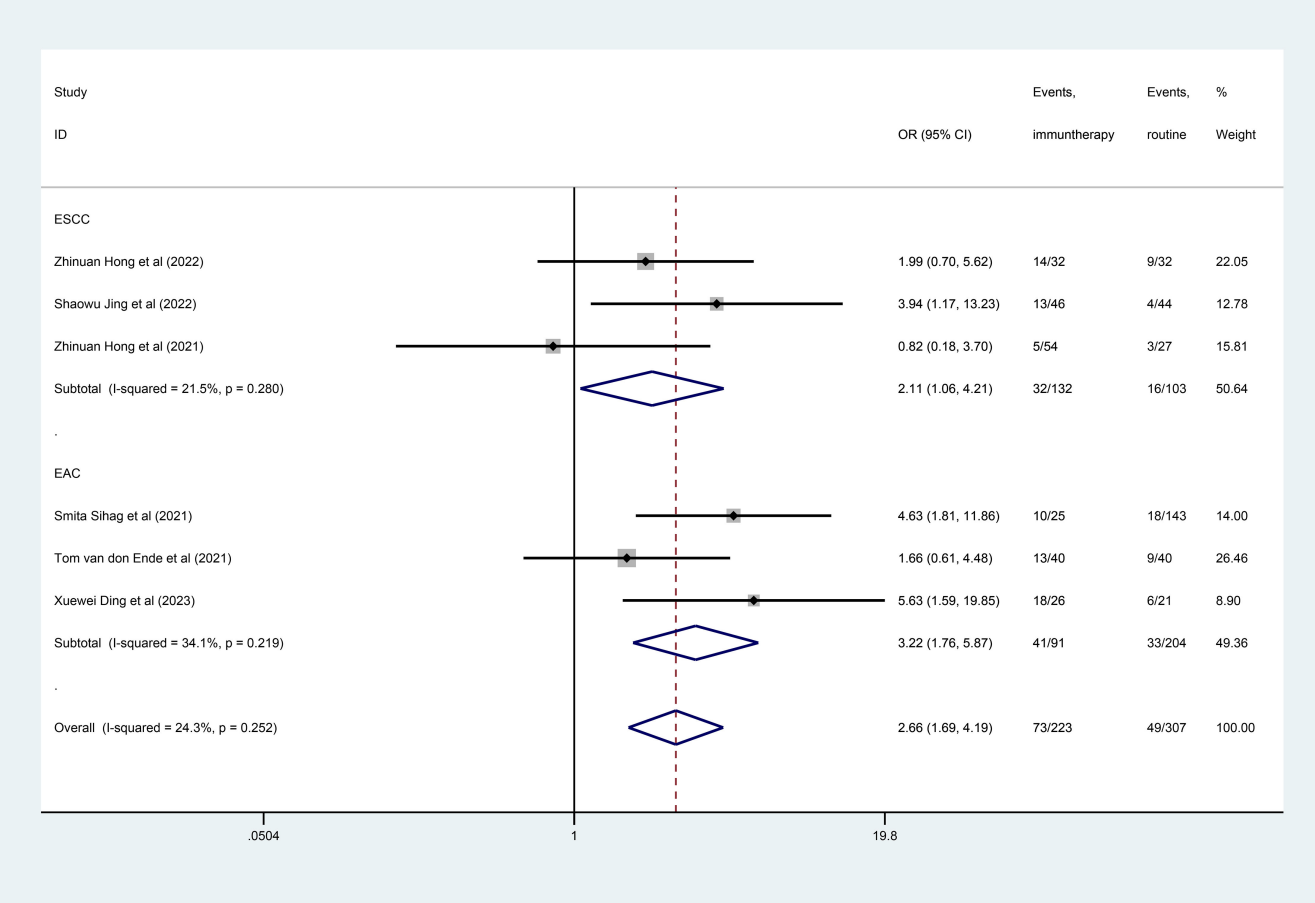
Forest plot of major pathological response (MPR).

Six studies (27–31, 33) reported on the MPR. When pooling the studies, the pooled MPR was higher in the neoadjuvant immunotherapy group (OR =2.66; 95% CI, 1.69–4.19; **Figure 3**). Common results were showed in the subgroups, EAC and ESCC. The result was showed in **Figure 3**.

### R0 resection

**Figure 4:**
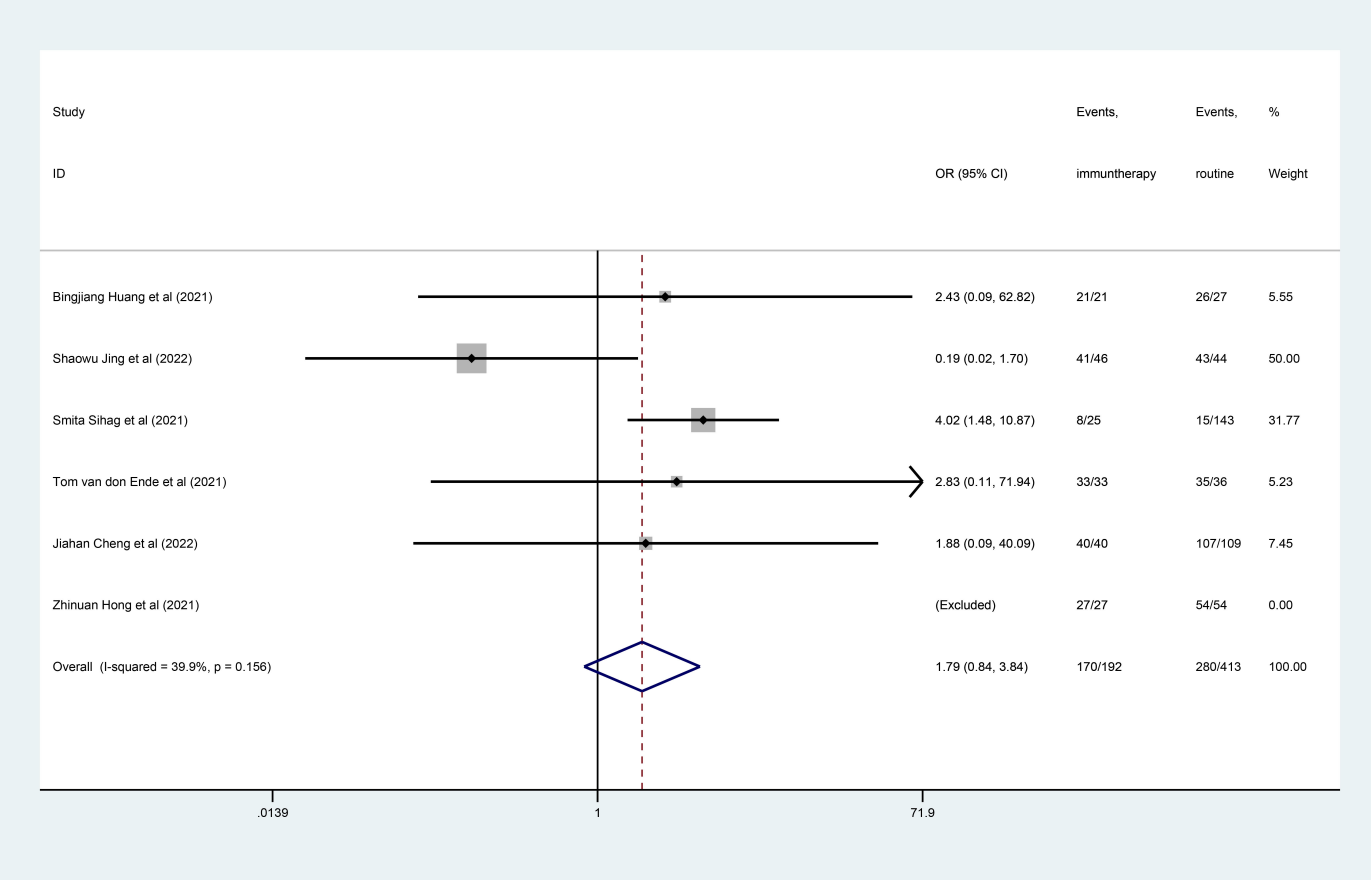
Forest plot of R0 resection.

No difference of R0 resection was founded between two groups (OR=1.79, 95% CI, 0.84–3.84; **Figure 4**), with moderate heterogeneity (I^2^ = 39.9%, *P*=0.156).

### Incidence of grade ≥3 TRAEs

**Figure 5:**
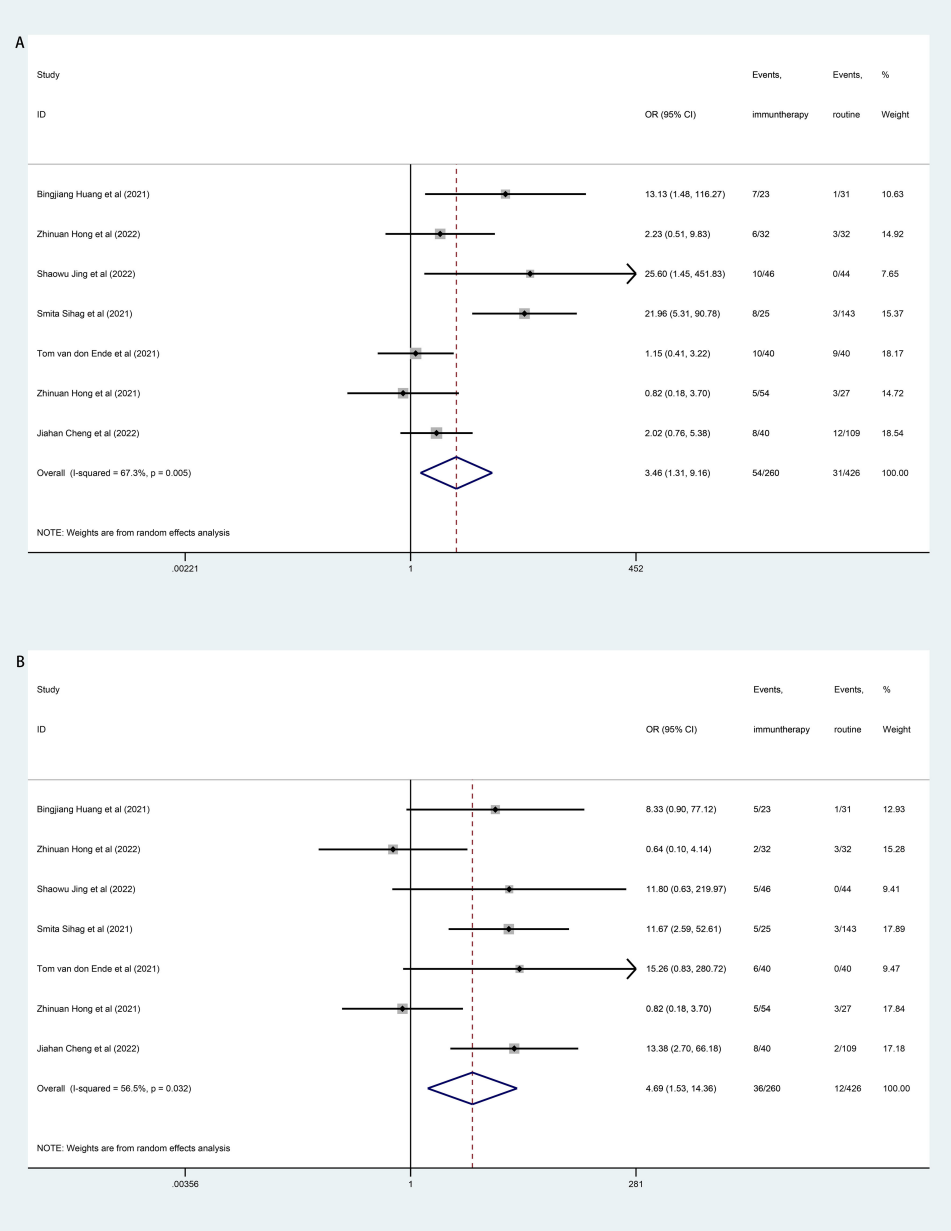
Forest plot of treatment related adverse events (TRAEs). **(A)**: Forest Plot of pneumonitis/pneumonia; **(B)**: Forest Plot of thyroid dysfunction.

Incidence of the overall grade ≥3 TRAEs was significantly higher in patients receiving neoadjuvant immunotherapy compared to patients receiving routine neoadjuvant therapy (neoadjuvant chemotherapy/chemoradiotherapy). Further analyses of individual grade ≥3 TRAEs showed that the neoadjuvant immunotherapy was associated with more pneumonitis/pneumonia (OR=3.46, 95% CI, 1.31–9.16; I^2^ = 67.3%, *P*=0.005; **Figure 5A**) and thyroid dysfunction (OR=4.69, 95% CI, 1.53–14.36; I^2^ = 56.5%, *P*=0.032; **Figure 5B**). Other individual grade ≥3 TRAEs including blood system, gastrointestinal system, and hypokalemia were comparable between the neoadjuvant immunotherapy and the routine neoadjuvant therapy (see **Supplementary Material 3: Figure S2**).

One death was reported in the patients received nICRT, and the death was due to pneumonitis (30).

### Surgical safety

Surgical resection rate (OR=0.74, 95% CI, 0.42–1.29; I^2^ = 0.0%, *P*=0.478) and surgical delay rate (OR=1.24, 95% CI, 0.79–1.90; I^2^ = 22.8%, *P*=0.255) were comparable between the neoadjuvant immunotherapy and the routine neoadjuvant therapy (see **Supplementary Material 3: Figure S3**). No surgical mortality was reported.

### Evaluation of sensitivity and publication bias

We conducted sensitivity analyses to ensure that the combined outcomes were not severely altered by the specific trials, and the overall estimates remained consistent across these analyses.

Egger’s test and Begg’s test were used to evaluate publication bias. Two regression intercept tests showed that the publication bias was not statistically significant (**Supplementary Material 3: Table S3**).

## Discussion

The neoadjuvant immunotherapy significantly improved pCR rates with tolerable toxicity in EC patients (14–17). However, the best neoadjuvant treatment strategy for EC was still inconclusive. Therefore, we conducted the comprehensive systematic review and meta-analysis to compare the antitumor efficacy and safety of the neoadjuvant immunotherapy with routine neoadjuvant therapy in patients with locally advanced EC. Our meta-analysis showed that the neoadjuvant immunotherapy had better pathologic response than routine neoadjuvant therapy. In addition, no significant differences were found in R0 resection rate.

The nCRT was performed as the standard therapy strategy for locally advanced EC patients, both ESCC and EAC. In the immune era, nCRT was also facing increasingly challenged by the neoadjuvant immunotherapy. The pembrolizumab combined with nCRT was demonstrated to be a safe and effective neoadjuvant treatment strategy for ESCC patients, in PALACE-1 trail. The neoadjuvant therapy did not delay surgery time, and 55.6% of patients received operation achieved pCR (34). Recent Neo-PLANET trail suggested that neoadjuvant camrelizumab plus nCRT exhibited pCR rate was 33.3% and MPR rate was 44.4% in patients with locally advanced EAC patients, with an acceptable safety profile. Although didn’t reach final survival outcome, Two-year progression free survival (PFS) and over survival (OS) rates were 66.9% and 76.1%, respectively (13). However, PERFECT trail suggested that the combining nCRT with immunotherapy didn’t show satisfactory database in patients with EAC (30). In addition, many trails also evaluated the clinical result of neoadjuvant immunotherapy in locally advanced EC patients, and the security of treatment was also analyzed (12, 35–40). The MPR and pCR for ESCC patients, received surgery, were 52.9%-72.0% and 30.2%-50.0% respectively. Preclinical studies have shown that programmed cell death 1 (PD-1) inhibitor combined with chemotherapy can further enhance the host’s immune response and inhibit the immune escape of cancer cells (41). For improving the efficacy, the neoadjuvant immunotherapy was always combined with chemotherapy or chemoradiotherapy (42).

Our study showed that the estimated pCR rates and MPR rates were higher in the neoadjuvant immunotherapy. But we found the pathologic response of the neoadjuvant immunotherapy appeared to be similar to that for nCRT in patients with locally advanced EC. At present, there were only two retrospective studies compared the antitumor efficacy and safety of nCRT with nICT. The study of Jiahan Cheng et al. indicated nICT could result in better outcome and less complications compared with nCRT therapy in locally advanced ESCC patients (32). However, Zhinuan Hong et al.’s study reported the quite opposite result (27). Platinum-based chemotherapy was the most applied neoadjuvant therapy. All included trails are based on the fluoropyrimidine plus platinum (FP) or the paclitaxel and carboplatin (PC). A three-arm phase III randomized controlled trial (JCOG1109) is ongoing in Japan (43); its preliminary results showed that the docetaxel, cisplatin plus 5-FU (DCF) would be a better choice. There was no consensus on the best chemotherapy regimen. In addition, the sequence of PD-1/PD-L1 inhibitors and chemotherapy or chemoradiotherapy might impact the pathologic response outcome. Wenqun Xing et al. found that delaying toripalimab to day 3 in nICT achieved a higher pCR rate, compared to on the same day (44). The time for surgical resection is generally 3-6 weeks after the last cycle neoadjuvant therapy. In our meta-analysis, 41.4Gy in was the most frequently used RT schedule in eligible studies of nICRT and nCRT.

There were no biomarkers could predicate clinical outcomes of the neoadjuvant immunotherapy for patients with EC. The most promising tools for predicting the potential for response to the neoadjuvant immunotherapy included PD-L1 expression status, mismatch-repair-deficient/microsatellite instability-high (dMMR/MSI-H), and tumor mutation burden (TMB). A recent meta-analysis suggested that tissue-based PD-L1 expression, more than any variable other than dMMR/MSI-H, identified varying degrees of benefit from ICIs-containing therapy (45). The dMMR/MSI-H also might be a biomarker (46). There was a strong association between TMB and clinical efficacy in advanced EAC patients received first-line pembrolizumab-based therapy, but it did not exclude patients with MSI-H tumors (47). A biomarker could accurately estimate the therapeutic effect of immunotherapy in esophageal cancer was eagerly needed.

Incidence rate of TRAEs was higher in the immunotherapy than routine neoadjuvant therapy. Our meta-analysis also suggested the same result, especially in pneumonitis/pneumonia and thyroid dysfunction. Tom van don Ende et al. reported one death due to pneumonitis (30); and dead cases caused by TRAEs were also reported in the PALACE-1 study (34). Unlike the TRAEs were within 10 days after the end of treatment in routine neoadjuvant therapy, TRAEs of immunotherapy usually occurred three and four weeks after one cycle of immunotherapy (48, 49). In addition, the danger of various TRAEs were totally different. Recent studies revealed that the TRAEs of skin and thyroid even were associated with a better prognosis (50).

### Limitations

There were several limitations in our study. Firstly, all included studies were descriptive study and the results have not been evaluated in large-scale controlled trials. Therefore, these findings required further validation by large RCTs. Only the RCTs were the golden standard of comparing the neoadjuvant immunotherapy and the routine neoadjuvant therapy. Secondly, researches for neoadjuvant immunotherapy in EAC remains fairly limited. The few researches were all performed in North America and Europe (29, 30). The diversity between ESCC and EAC might may lead to different responses to the neoadjuvant immunotherapy. Therefore, more clinical trials of neoadjuvant immunotherapy in EAC are needed, especially in East Asia. The main outcome measures are pCR and MPR, both would be typically increased by radiotherapy. A clear comparison between nICT vs nCT and nICRT vs nCRT is not achievable for the smaller sample size of the included studies. Thirdly, all eligible studies concentrated the pathological response rates, but no survival data was reported. The association between pathological response and survival in esophageal cancer deserves further investigation (51). Only the overall survival data was the gold standard to compare the neoadjuvant immunotherapy with routine neoadjuvant therapy. Another main limitation is the heterogeneity of the included studies, which is reflected in the different ICIs.

## Conclusions

The current meta-analysis revealed that the neoadjuvant immunotherapy (nICT and nICRT) could significantly increase the rates of pCR and MPR, compared with routine neoadjuvant therapy (nCT and nCRT) in the treatment of locally advanced EC. The neoadjuvant immunotherapy and routine neoadjuvant therapy were with acceptable toxicity. However, randomized studies with larger groups of patients need to performed to confirm these results.

## Data availability statement

The original contributions presented in the study are included in the article/**Supplementary Material**. Further inquiries can be directed to the corresponding author.

## Author contributions

FL: conceptualization. HX and KL: methodology. HQ and HX: software. YL: formal analysis. HQ, HX and KL: data curation. YZ: writing original draft preparation. YZ, LW, HQ and FY: writing-review and editing. All authors contributed to the article and approved the submitted version.
