# Supplementary material for: Comparison of neoadjuvant immunotherapy versus routine neoadjuvant therapy for patients with locally advanced esophageal cancer: A systematic review and meta-analysis
Source: Front Immunol. 2023 Mar 23;14:1108213. doi: 10.3389/fimmu.2023.1108213 (PMC10076616; doi:10.3389/fimmu.2023.1108213)
Supplement: Supplementary file 1 [file DataSheet_1.pdf]

Supplementary materials

Table S1. PubMed search strategy.

Table S2. The Newcastle-Ottawa scale (NOS) quality assessment of the enrolled studies.

Table S3. Result of meta-regression analyses on pCR and MPR.

Figure S1. Subgroups of neoadjuvant immune checkpoint inhibitor in combination with chemotherapy

Figure S2. Forest Plot. A) blood system; B) gastrointestinal system; C) hypokalemia.

Figure S3. Forest Plot. A) surgical resection rate; B) surgical delay rate.

Table S1. PubMed search strategy.

# No	Searches
Part I: Immune checkpoint inhibitor	
**1**	“Programmed Cell Death 1 Receptor”[Mesh] OR “CTLA-4 Antigen”[Mesh]
**2**	“anti-PD-1”[Title/Abstract] OR “PD-1”[Title/Abstract] OR “anti-PD-L1”[Title/Abstract] OR “PD-L1”[Title/Abstract] OR “anti-PD(L)-1”[Title/Abstract] OR “PD(L)-1”[Title/Abstract] OR “CTLA-4”[Title/Abstract] OR “anti-CTLA-4”[Title/Abstract] OR ” anti-cytotoxic T-lymphocyte antigen-4”[Title/Abstract]
**3**	“nivolumab”[Title/Abstract] OR “pembrolizumab”[Title/Abstract] OR “atezolizumab”[Title/Abstract] OR “durvalumab”[Title/Abstract] OR “avelumab”[Title/Abstract] OR “ipilimumab”[Title/Abstract] OR “cemiplimab”[Title/Abstract]
**4**	“immune checkpoint inhibitor”[Title/Abstract] OR “immune checkpoint inhibitors”[Title/Abstract] OR “ICI”[Title/Abstract] OR “immune checkpoint blockade”[Title/Abstract] OR “ICB”[Title/Abstract]
**5**	1 or 2 or 3 or 4
Part II: Esophageal cancer	
**6**	“Esophageal Neoplasm”[Title/Abstract] OR “Esophagus Neoplasm”[Title/Abstract] OR “esophageal adenocarcinoma”[Title/Abstract] OR “esophageal squamous cell carcinoma”[Title/Abstract] OR “Esophagus Cancer”[Title/Abstract]
Part III: Neoadjuvant therapy	
**7**	“Neoadjuvant Therapies”[Title/Abstract] OR “Neoadjuvant Treatment”[Title/Abstract] OR “Neoadjuvant Radiotherapy”[Title/Abstract] OR “Neoadjuvant Radiation Treatment”[Title/Abstract] OR “Neoadjuvant Systemic Therapy”[Title/Abstract] OR “Neoadjuvant Chemotherapy”[Title/Abstract] OR “Neoadjuvant Chemoradiotherapies”[Title/Abstract] OR “Neoadjuvant Chemoradiation Treatment”[Title/Abstract] OR “Neoadjuvant Chemoradiation Therapy”[Title/Abstract]
Part IV: Part I, II, AND III	
**8**	5 and 6 and 7

Table S2. The Newcastle-Ottawa scale (NOS) quality assessment of the enrolled studies.

Study ID	SELECTION				COMPARABILITY	OUTCOME			Total[a]
	Representativeness of the exposed cohort	Selection of the nonexposed cohort	Ascertainment of exposure	Demonstration that outcome of interest was not present at start of study[b]	Comparability of cohorts on the basis of the design or analysis[c]	Assessment of outcome	Was follow-up long enough for outcomes to occur[d]	Adequacy of follow up of cohorts[e]	
Bingjiang Huang et al, 2021	truly*	same institute*	record*	yes*	*	record*	no	not clear	6
Zhinuan Hong et al, 2022	truly*	same institute*	record*	no	*	record*	no	not clear	5
Shaowu Jing et al, 2022	truly*	same institute*	record*	no	*	record*	yes*	yes*	7
Smita Sihag et al, 2021	truly*	same institute*	record*	no	-	record*	yes*	yes*	6
Tom van don Ende et al, 2021	truly*	same institute*	record*	no	*	record*	no	yes*	6
Zhinuan Hong et al, 2021	somewhat*	same institute*	record*	no	*	record*	yes*	yes*	7
Jiahan Cheng et al, 2022	truly*	same institute*	record*	no	-	record*	yes*	yes*	6

Xuewei Ding et al, 2023	somewhat*	same institute*	record*	no	*	record*	yes*	yes*	7

- indicates Zero score, * indicates one score, ** indicates two scores

Variables	pCR			MPR		
	Cofe.	Std. Err.	P value	Cofe.	Std. Err.	P value
Year	-0.7282	0.5035	0.2080	-0.1780	0.2679	0.4965
Country	0.0010	0.2415	0.9960	-0.6597	0.5443	0.2005
Patient no.	0.0354	0.6080	0.9554	-0.8783	0.5981	0.1850
strategy	0.0110	0.3765	0.8329	-2.1563	0.2256	0.0008
Quality	0.0236	0.5163	0.8224	-1.7562	0.3264	0.0020

Table S3. Result of meta-regression analyses on pCR and MPR.

pCR: Pathologic complete response

MPR: major pathological response

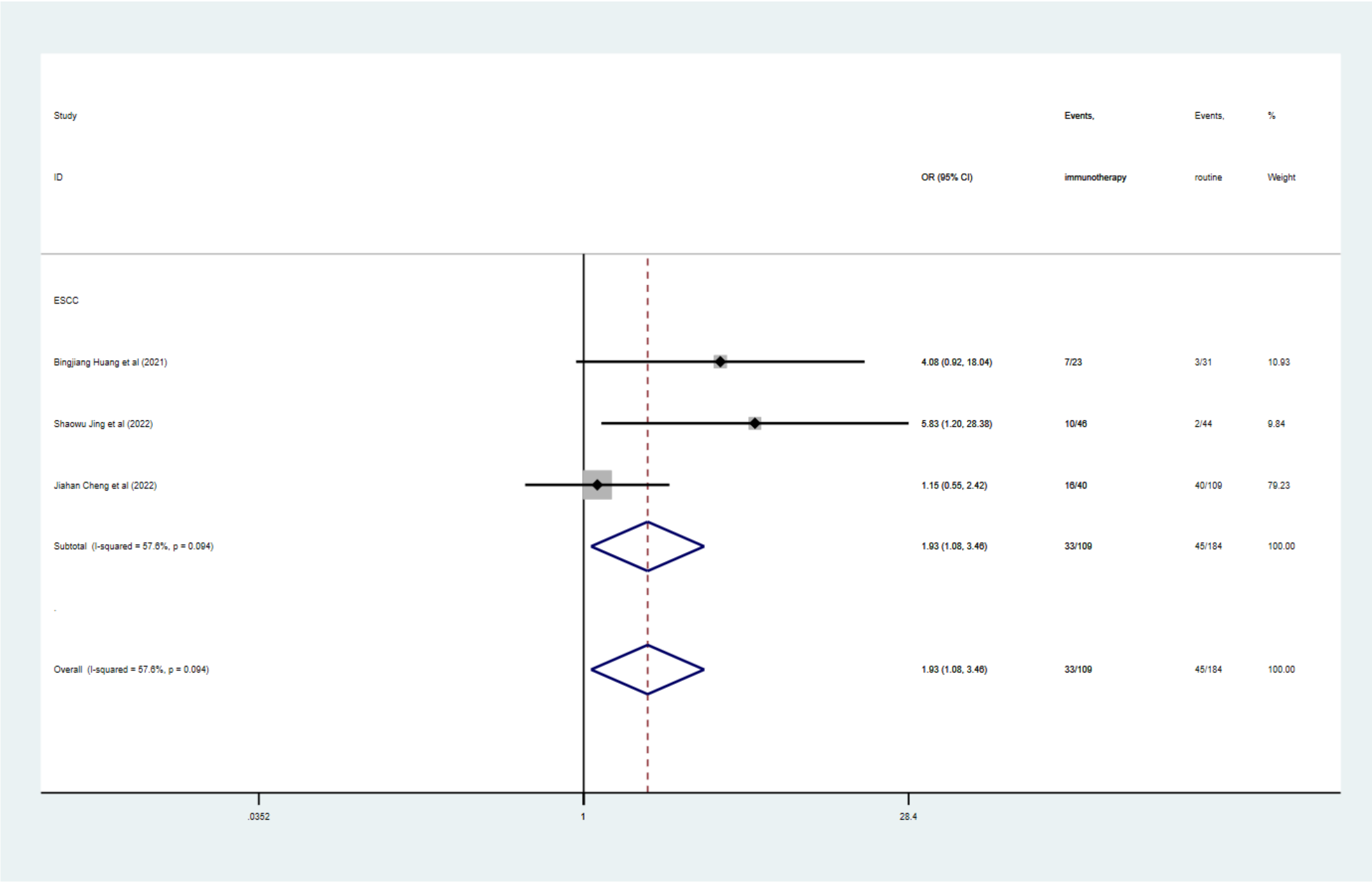

Figure S1. Subgroups of neoadjuvant immune checkpoint inhibitor in combination with chemotherapy

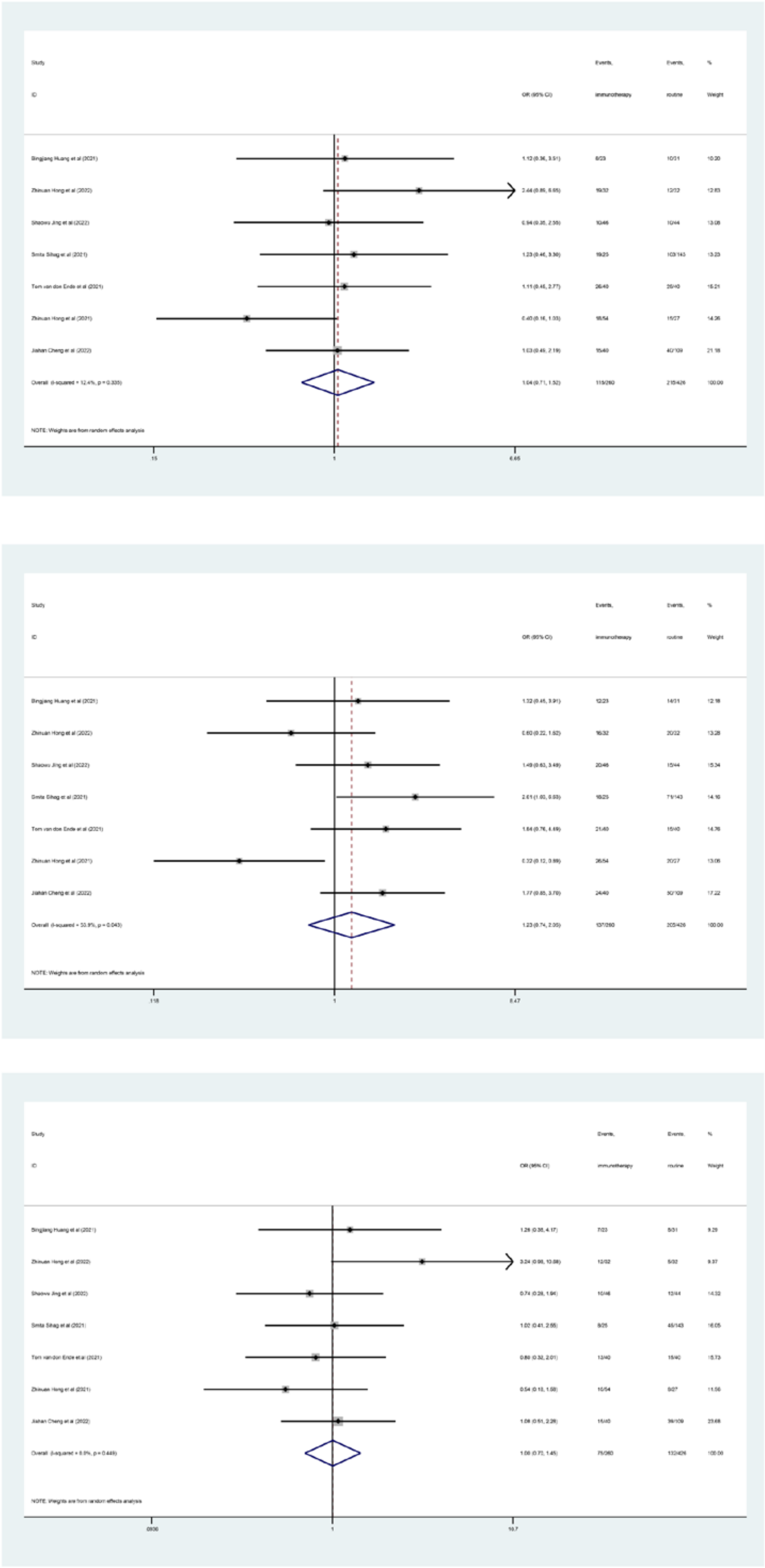

A
B
C
Study
ID
OR (95% CI)
Events, immunotherapy
Events, routine
% Weight
Bingjiang Huang et al (2021)
Zhinuan Hong et al (2022)
Shaowu Jing et al (2022)
Smita Sihag et al (2021)
Tom van den Ende et al (2021)
Zhinuan Hong et al (2021)
Jiahan Cheng et al (2022)
Overall
NOTE: Weights are from random effects analysis

Figure S2. Forest Plot. A) blood system; B) gastrointestinal system; C) hypokalemia.

A

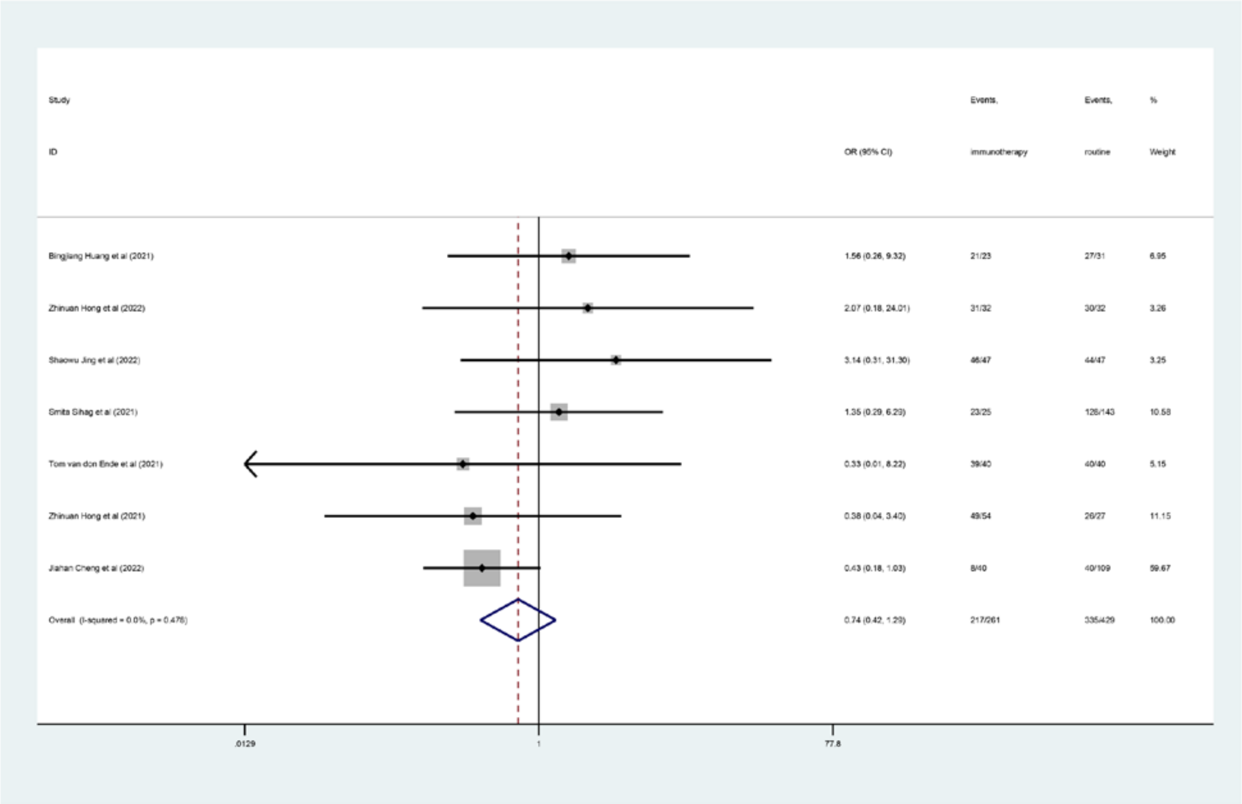

B

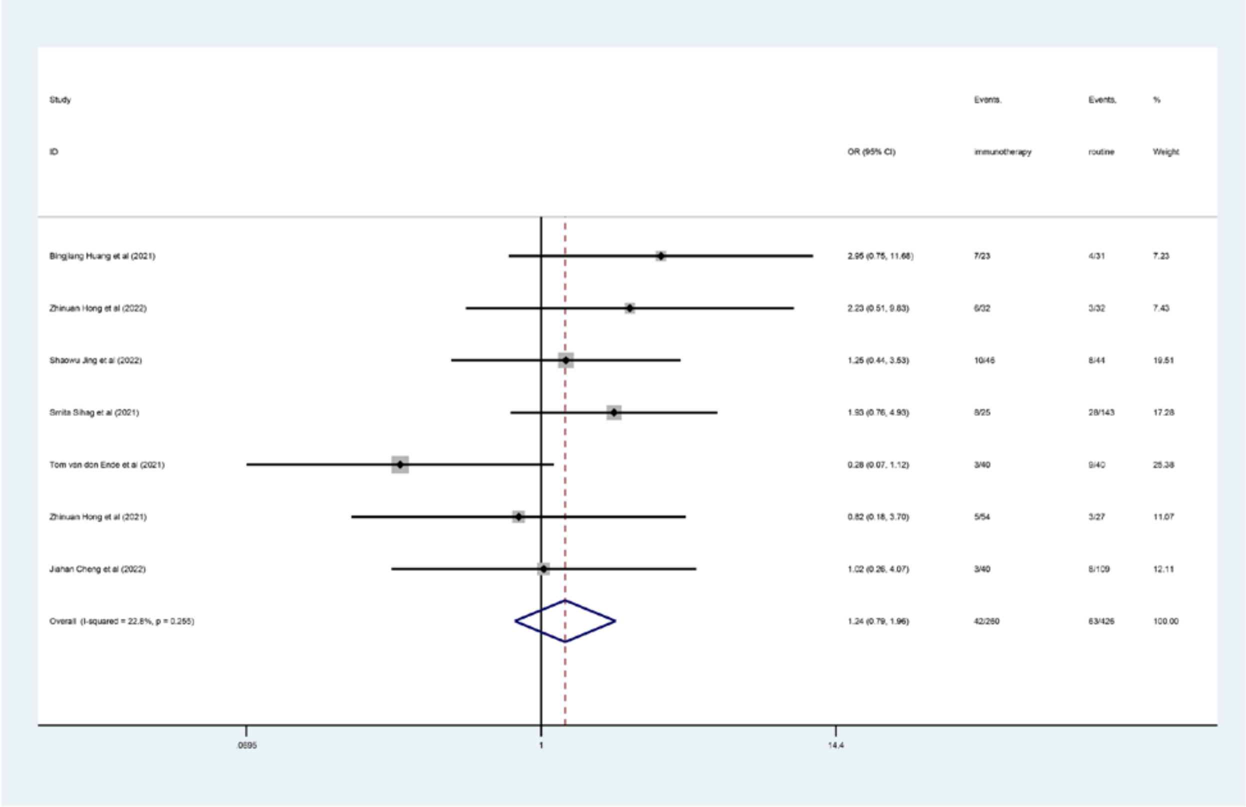

Figure S3. Forest Plot. A) surgical resection rate; B) surgical delay rate.
